# Does pharmacist-supervised intervention through pharmaceutical care program influence direct healthcare cost burden of newly diagnosed diabetics in a tertiary care teaching hospital in Nepal: a non-clinical randomised controlled trial approach

**DOI:** 10.1186/s40199-016-0145-x

**Published:** 2016-02-29

**Authors:** Dinesh Kumar Upadhyay, Mohamed Izham Mohamed Ibrahim, Pranaya Mishra, Vijay M. Alurkar, Mukhtar Ansari

**Affiliations:** Faculty of Pharmacy, Asian Institute of Medicine, Science and Technology University, Jalan Bedong-Semeling, 08100 Bedong, Kedah Malaysia; Social and Administrative Pharmacy, College of Pharmacy, Qatar University, 2713 Doha, Qatar; Department of Pharmacology, American University of the Caribbean School of Medicine, 1 University Drive at Jordan Road, Cupecoy, St. Maarten, Netherlands Antilles; Department of Medicine, Manipal College of Medical Sciences and Manipal Teaching Hospital, Phulbari-11, Pokhara, Nepal; Department of Pharmacy and Pharmacology, National Medical College, Birgunj, Nepal

**Keywords:** Diabetes mellitus, Healthcare costs, Intervention, Nepal, Pharmacists, Pharmaceutical care, Randomised controlled trial

## Abstract

**Background:**

Cost is a vital component for people with chronic diseases as treatment is expected to be long or even lifelong in some diseases. Pharmacist contributions in decreasing the healthcare cost burden of chronic patients are not well described due to lack of sufficient evidences worldwide. In developing countries like Nepal, the estimation of direct healthcare cost burden among newly diagnosed diabetics is still a challenge for healthcare professionals, and pharmacist role in patient care is still theoretical and practically non-existent. This study reports the impact of pharmacist-supervised intervention through pharmaceutical care program on direct healthcare costs burden of newly diagnosed diabetics in Nepal through a non-clinical randomised controlled trial approach.

**Methods:**

An interventional, pre-post non-clinical randomised controlled study was conducted among randomly distributed 162 [control (*n* = 54), test 1 (*n* = 54) and test 2 (*n* = 54) groups] newly diagnosed diabetics by a consecutive sampling method for 18 months. Direct healthcare costs (direct medical and non-medical costs) from patients perspective was estimated by ‘bottom up’ approach to identify their out-of-pocket expenses (1USD = NPR 73.38) before and after intervention at the baseline, 3, 6, 9 and 12 months follow-ups. Test groups’ patients were nourished with pharmaceutical care intervention while control group patients only received care from physician/nurses. Non-parametric tests i.e. Friedman test, Mann–Whitney *U* test and Wilcoxon signed rank test were used to find the differences in direct healthcare costs among the groups before and after the intervention (*p* ≤ 0.05).

**Results:**

Friedman test identified significant differences in direct healthcare cost of test 1 (*p* < 0.001) and test 2 (*p* < 0.001) groups patients. However, Mann–Whitney *U* test justified significant differences in direct healthcare cost between control group and test 1 group, and test 2 group patients at 6-months (*p* = 0.009, *p* = 0.010 respectively), 9-months (*p* = 0.005, *p* = 0.001 respectively) and 12-months (*p* < 0.001, *p* < 0.001 respectively).

**Conclusion:**

Pharmacist supervised intervention through pharmaceutical care program significantly decreased direct healthcare costs of diabetics in test groups compared to control group and hence describes pharmacist’s contribution in minimizing direct healthcare cost burden of patients.

## Background

Cost is a vital component for people with chronic diseases as treatment is expected to be long, even life-long in many cases. Pharmacist contributions in decreasing the healthcare cost burden of chronic patients are not well described due to lack of sufficient evidences worldwide. Direct healthcare costs (DHCs) are the “actual monetary expenditure used in treating or coping with a disease” [1]. In a broader sense, the direct healthcare cost is the expenditure spent for detection, treatment, rehabilitation and care of a disease [2]. Distinguishing the definition of cost from price, “cost is a function of the inputs (labour, consumable goods, depreciation, etc.) required to produce a particular service” while “price is a function of what is paid in the market place” [3]. Due to the chronic nature of diabetes, it is associated with a substantial impact on the healthcare cost of patients^2^with the largest proportion attributed to treatment cost among other components of direct cost of diabetes [1, 4]. The global estimation of direct healthcare expenditure for people with diabetes is about USD 153 billion per year [5].

In Nepal, where healthcare services are poor [6] and not streamlined, people have difficulty in accessing the healthcare services most of the time. Furthermore, in absence of government and private healthcare insurance coverage, patients pay from their pocket to avail the acquired healthcare services, which increase their out-of-pocket expenses and make the treatment unaffordable to them. This result in a delay in disease diagnosis and early episodes of complications that may lead to frequent hospitalization and increased prescription cost with subsequent effects on other components of the direct cost domain [7]. Pharmacist role in patient care is still theoretical and practically non-existent in Nepal resulting in huge healthcare cost burden on patients. This study reports the impact of pharmacist-supervised intervention through pharmaceutical care program on direct healthcare costs of newly diagnosed diabetics in Nepal.

## Methods

### Study design

An interventional, pre-post non-clinical randomised controlled trial among the control group (CG), test 1 group (T1G) and test 2 group (T2G) with three treatment arms was conducted to explore the impact of pharmacist-supervised pharmaceutical care intervention on direct healthcare cost of newly diagnosed diabetics at the Manipal Teaching Hospital, Pokhara, Nepal for 18 months (July 2010 to December 2011). The study was approved by the Research and Ethics Committee of Manipal Teaching Hospital, Nepal [8].

### Study population

Newly diagnosed type 1 and type 2 diabetes mellitus patients of aged 16 years and above were selected. Pregnant women, mentally incompetent patients, patients not willing to participate and did not come at their first follow-up were excluded from the study. Written consent was taken from patients participating in the study. However, in case of minors, parental consent was sought and obtained.

### Sample and sampling technique

Sample size was calculated by using a finite population correction formula [9]. Diabetes prevalence of 9 % was taken as the calculation factor from previous studies [10, 11]. The Z value was set at 1.96, with a 95 % of confidence interval and 5 % as margin of error. The calculated sample size was 125 patients. A drop-outs margin of 30 % was taken from previous studies [12, 13] and added to the sample to achieve the final targeted sample size of 162 patients. The targeted sample was achieved by a consecutive sampling method (based on time capsule frame) over 6 months duration (July 2010 to December 2010) [14]. The randomisation of 162 patients was done by 1:1:1 in three parallel groups [CG (*n* = 54), T1G (*n* = 54) and T2G (*n* = 54)] without disturbing the sequence of randomisation [15]. Ten patients (CG = 4; T1G = 3 and T2G = 3) did not complete their first assessment follow-up (3-months) and therefore, further study was carried out with 152 patients [CG (*n* = 50), T1G (*n* = 51) and T2G (*n* = 51)] [8].

### Study tools

Study tools were prepared in the Nepali language due to language fluency and barriers to the English language among most of the patients visiting the hospital. Socio-demography form was used to collect the patients’ demographic characteristics. Direct healthcare costs documentation form was used to analyse the cost incurred by the patients in diabetes management during study period. Diabetes information booklet, diabetes complication chart and diabetic food chart were educational materials to improve the patients’ awareness about diabetes and its management. A diabetic kit (including glass tubings, chart of human anatomy with circulatory system, daily medication calendar and calendar of anti-diabetic medicines) was made especially for T2G (PC + Diabetic kit group) patients to explain about anatomical and physiological relationship of diabetes and its impact on physiological system. The intention to use diabetic kit only in T2G patients was to identify whether an extra demonstration of diabetic kit would increase patient’s understanding about diabetes and assist them for better disease control. This extra initiative might bring remarkable differences in direct healthcare cost burden of patients between T1G and T2G [8].

### Estimation of direct medical and non-medical costs of patients

The direct healthcare costs estimation (direct medical and non-medical costs) from the patient’s perspective was done by a ‘bottom up’ approach [16] to calculate their out-of-pocket expenses in managing their diabetes during the study period (12-months). The ‘bottom-up’ approach is based on the cost of individual units of service provided. The ‘bottom-up’ approach in fact starts from a selected subpopulation with the actual disease and all the costs associated with the disease is collected and extrapolated to the national level [17].

Patient’s direct healthcare costs were estimated before and after pharmacist-supervised pharmaceutical care intervention at the baseline and 3, 6, 9 and 12 months’ follow-ups respectively. Direct healthcare costs incurred by patients at baseline and each follow-up are the total sum of direct medical and non-medical costs. Direct medical costs comprised of various cost-variables including patient registration cost, cost for emergency care, lab investigation cost, drug(s) cost and cost of hospitalization and in-patient care. However, direct non-medical costs include transportation cost, meal cost on the way to hospital [1, 18] and dietary management cost during investigation in hospital. Information related to direct medicals cost was taken from patient’s medical record (bills and prescriptions, etc.) and hospital rate lists for different services.

To calculate direct medical costs on patient is multiplying the number of each service/care provided by the unit cost of each service/care. For laboratory investigations, the number of laboratory tests performed was multiplied by the unit cost of each test. However, the calculation of drug cost(s) was done by multiplying the number of dose by the unit price of the drug and the resulting total costs was then multiplied by duration of therapy to obtain the total drug costs. However, direct non-medical cost estimation was done on the basis of information collected from patients and their relatives with regard to transportation cost, meal cost on the way to hospital (to and fro) and dietary management cost on each visit to the hospital.

Patients were asked to maintain a copy of all the bills and prescriptions related to their treatment to ensure the maximum accuracy of cost estimation. The calculation of DHCs at each follow-up covered direct medical and non-medical costs of patient between the two follow-ups (e.g. direct healthcare costs at 3-months will be the sum of direct medical and non-medical costs between baseline and 3-months period and so on). All the information related to direct healthcare costs were documented in a pre-designed direct healthcare cost documentation form.

### Pharmacist intervention among diabetes patients

Pharmacist had made an attempt to minimize direct healthcare cost burden of diabetics by improving their understanding about diabetes. Pharmacist led intervention was done among the patients of test groups (T1G and T2G). Education and counselling about different aspects of diabetes and its management and, the correct use of antidiabetic medications were the important information covered by the pharmacist during the intervention. Patients from the test groups received the information about meaning of diabetes, its types, sign and symptoms, reasons for high blood glucose, risk factors of diabetes, different short term and long term complications of diabetes and role of pharmacological (anti-diabetic medication) and non-pharmacological (lifestyle modification, diet and exercise) measures in management of diabetes from pharmacist. Besides this, test groups patients were also taught about how to administer insulin by using insulin pen or insulin syringe (if insulin was prescribed in therapy) and trained regarding the use of glucometer for self-monitoring of blood glucose (SMBG) at home. Medication envelopes were used to dispense the prescribed medication (s) to the patients.

In addition to it, the test 2 group patients received the demonstration of diabetic kit components such as glass tubing’s showing the change in the viscosity pattern of blood among diabetic and non-diabetic patients and the impact of increased sugar on the blood flow in different organ system with emphasis of blood coagulation and obstruction in blood flow in blood vessels in diabetes. Chart of human anatomy with circulator system was described to make the patients aware to locate the different organ system in the body and the supply of blood to these organs via blood vessels. Special focus was given to those organs which are mainly affected in diabetes i.e. cardiac system, renal system, eye and brain. They were also explained about the location of pancreas and its role in diabetes. Daily medication calendar and anti-diabetic medicine calendar were used to enhance the patients’ knowledge and compliance about the use of anti-diabetic medication in diabetes management [8].

### Statistical analysis

The direct healthcare costs of patients from three groups were calculated at the baseline, 3, 6, 9 and 12 months’ follow-ups. Data was entered in SPSS version 16 and descriptive analysis was done as required for data analysis. Data was skewed (*p* < 0.05) on Kolmogorov-Smirnov test. Non-parametric tests i.e. Friedman test, Mann–Whitney *U* test were used to find out the differences between dependent and independent variables within and between the groups before and after the interventions respectively. The Wilcoxon signed rank test was used for pre- and post-comparison within the groups. Post hoc analysis with Wilcoxon signed rank test was used to find out in which follow-up the significant differences actually occurred in the group at a new *p*-value of ≤0.005 after Bonferroni adjustment. A significance level of *p* ≤ 0.05 was used in all analyses.

## Results

### Socio-demography of patients

The study enrolled 162 patients. The mean age (in years) of the patients was 49.14 ± 12.56. Males were greater in number (*n* = 106, 65.43 %). The median monthly income and inter-quartile range of the patients was Nepali rupees (NPR) 10,000 [(9,000)-(16,000)] (1USD = 73.38 NPR). About 40.7 % patients were unemployed, 25.9 % businessman, 18.5 % employed, 13.6 % pensioner and 1.2 % students in the study. The study found 30.9 % patients either primary educated or secondary educated and, only 24 % and 14.2 % patients were non-educated and tertiary educated respectively. There were no significant differences in education level and health related knowledge among the patients of three groups at baseline. There were 92 % patients of non-vegetarian food habits. Nearly 42.6 % and 57.4 % patients never had alcohol and smoking habits respectively. Type 2 diabetics were found more (*n* = 156, 96.3 %) in the study [8].

### Geometric changes in direct medical and non-medical costs of CG, T1G and T2G patients at the baseline and follow-ups

Descriptive analysis was done to calculate direct medical and non-medical costs burden on diabetics and results are presented in mean ± sd and median (IQR) cost. The chief contributors of direct medical and non-medical costs of the control and test groups’ patients were cost of investigation, drug(s) costs, patient registration cost, and transportation cost, dietary management cost respectively. Pharmacist-provided intervention reduced direct medical and non-medical cost burden on patients in test groups with greater reduction in anti-diabetic treatment cost in subsequent follow-ups (Table 1).

**Table 1:** Geometric changes in direct medical and non-medical costs of CG, T1G, and T2G patients at the baseline and follow-ups^a^

Direct medical cost												
Cost variables		Groups^b^	Baseline		3-months (1^st^ FU)		6-months (2^nd^ FU)		9-months (3^rd^ FU)		12-months (4^th^ FU)	
			Mean cost ± sd	Median cost (IQR)	Mean cost ± sd	Median cost (IQR)	Mean cost ± sd	Median cost (IQR)	Mean cost ± sd	Median cost (IQR)	Mean cost ± sd	Median cost (IQR)
Patient registration		CG	30.00 ± .00	30 (30)-(30)	76.20 ± 29.82	60 (60)-(90)	94.80 ± 22.15	90 (90)-(120)	81.00 ± 34.41	90 (60)-(97.50)	90.60 ± 22.26	90 (90)-(90)
		T1G	30.00 ± .00	30 (30)-(30)	102.94 ± 20.12	90 (90)-(120)	66.47 ± 19.26	60 (60)-(90)	50.58 ± 20.33	60 (30)-(60)	49.41 ± 20.63	60 (30)-(60)
		T2G	30.00 ± .00	30 (30)-(30)	69.41 ± 23.61	60 (60)-(90)	67.05 ± 22.28	60 (60)-(90)	53.52 ± 20.18	60 (30)-(60)	41.17 ± 17.96	30 (30)-(60)
Emergency care (if any)		CG T1G T2G	0	0	0	0	0	0	0	0	0	0
Hospitalization and in-patient care (if any)		CG	51.12 ± 264.08	0 (0)-(0)	0	0	16.00 ± 113.13	0 (0)-(0)	0	0	0	0
		T1G	27.48 ± 139.55	0 (0)-(0)	0	0	0	0	0	0	0	0
		T2G	19.44 ± 142.88	0 (0)-(0)	0	0	0	0	0	0	0	0
Investigations		CG	406.20 ± 222.35	350 (313.25)-(430)	464.60 ± 92.45	450 (400)-(542.50)	462.88 ± 89.96	450 (440)-(492.50)	445.46 ± 104.63	400 (400)-(455)	459.50 ± 114.59	450 (400)-(500)
		T1G	413.29 ± 161.34	350 (340-460)	474.90 ± 88.20	455 (450)-(500)	425.92 ± 72.33	405 (400)-(450)	398.07 ± 72.60	400 (355)-(405)	385.05 ± 56.29	355 (350)-(400)
		T2G	399.75 ± 116.60	350 (340)-(405)	435.49 ± 91.76	400 (400)-(450)	417.90 ± 82.49	400 (390)-(450)	398.68 ± 53.69	400 (355)-(405)	367.35 ± 34.19	350 (350)-(400)
Drug (s) cost^c^	ADD	CG	211.40 ± 226.82	109 (45)-(304.75)	1080.00 ± 869.36	870 (560)-(1236.50)	869.48 ± 435.99	880 (584)-(995)	755.86 ± 302.69	760 (560)-(978.50)	661.28 ± 294.93	650 (495)-(884.70)
	AntiHTN		50.14 ± 101.61	0 (0)-(77)	148.66 ± 366.41	0 (0)-(182)	250.34 ± 398.26	0 (0)-(650)	248.00 ± 310.34	0 (0)-(560)	253.58 ± 306.02	0 (0)-(540.75)
	Others		132.12 ± 496.84	0 (0)-(73.50)	157.16 ± 541.23	0 (0)-(13.50)	182.06 ± 358.23	0 (0)-(91.75)	164.52 ± 389.07	0 (0)-(67.75)	154.90 ± 390.06	0 (0)-(54)
	ADD	T1G	216.75 ± 274.08	141 (47)-(316)	993.19 ± 838.77	761 (288)-(1544)	790.49 ± 840.25	480 (0)-(1222)	665.27 ± 538.23	508 (284)-(929)	436.21 ± 534.41	254 (48)-(692)
	AntiHTN		33.11 ± 76.89	0 (0)-(6.25)	169.15 ± 353.51	0 (0)-(222)	166.39 ± 389.02	0 (0)-(0)	114.62 ± 234.93	0 (0)-(0)	84.64 ± 195.71	0 (0)-(0)
	Others		82.85 ± 153.45	0 (0)-(110.75)	250.76 ± 720.28	0 (0)-(0)	172.31 ± 579.29	0 (0)-(0)	148.54 ± 506.22	0 (0)-(0)	121.29 ± 360.94	0 (0)-(0)
	ADD	T2G	158.42 ± 186.14	83.50 (20.50)-(212)	1035.80 ± 669.29	1015 (480)-(1500)	754.33 ± 856.70	350 (48)-(1269)	579.00 ± 798.76	367 (139)-(750)	315.47 ± 470.88	157 (0)-(413)
	AntiHTN		33.48 ± 90.07	0 (0)-(0)	178.00 ± 309.62	0 (0)-(215)	121.29 ± 218.61	0 (0)-(277)	91.45 ± 210.78	0 (0)-(0)	73.07 ± 180.94	0 (0)-(0)
	Others		64.70 ± 207.71	0 (0)-(0)	192.66 ± 509.89	0 (0)-(0)	151.86 ± 386.75	0 (0)-(54)	154.07 ± 536.24	0 (0)-(0)	112.96 ± 379.02	0 (0)-(0)
Direct non-medical cost												
Cost variables		Groups^b^	Baseline		3-months (1^st^ FU)		6-months (2^nd^ FU)		9-months (3^rd^ FU)		12-months (4^th^ FU)	
			Mean cost ± sd	Median cost (IQR)	Mean cost ± sd	Median cost (IQR)	Mean cost ± sd	Median cost (IQR)	Mean cost ± sd	Median cost (IQR)	Mean cost ± sd	Median cost (IQR)
Transport (round trips)		CG	166.53 ± 314.99	54 (30)-(162.50)	233.36 ± 196.70	155 (87.50)-(312.50)	112.34 ± 53.20	120 (69)-(150)	101.60 ± 49.29	100 (70)-(140)	125.32 ± 71.84	120 (78.75)-(165)
		T1G	110.38 ± 277.22	30 (24)-(85)	108.62 ± 62.76	100 (80)-(150)	117.54 ± 98.23	80 (50)-(160)	109.80 ± 100.45	80 (40)-(150)	108.33 ± 95.28	80 (48)-(120)
		T2G	100.09 ± 176.45	40 (28.75)-(70)	116.82 ± 64.04	120 (60)-(200)	117.39 ± 77.31	100 (60)-(160)	113.07 ± 113.07	80 (48)-(120)	117.98 ± 154.49	80 (40)-(108)
Meal on the way to hospital (round trip, if any)		CG	1.85 ± 13.60	0 (0)-(0)	11.70 ± 29.06	0 (0)-(0)	6.24 ± 22.65	0 (0)-(0)	8.60 ± 24.05	0 (0)-(0)	7.10 ± 21.33	0 (0)-(0)
		T1G	3.70 ± 27.21	0 (0)-(0)	8.56 ± 27.75	0 (0)-(0)	6.54 ± 18.77	0 (0)-(0)	5.86 ± 20.77	0 (0)-(0)	3.54 ± 10.07	0 (0)-(0)
		T2G	2.22 ± 16.32	0 (0)-(0)	7.05 ± 20.20	0 (0)-(0)	3.88 ± 11.20	0 (0)-(0)	7.01 ± 23.35	0 (0)-(0)	7.25 ± 23.18	0 (0)-(0)
Dietary management during investigation		CG	137.37 ± 293.93	60 (50)-(100)	147.00 ± 44.82	150 (120)-(200)	183.20 ± 100.21	180 (140)-(200)	157.50 ± 52.37	160 (120)-(200)	172.00 ± 30.10	170 (150)-(200)
		T1G	111.12 ± 191.28	60 (60)-(80)	142.15 ± 34.13	150 (120)-(160)	123.03 ± 47.46	120 (80)-(150)	111.07 ± 59.27	90 (80)-(120)	114.31 ± 47.17	100 (80)-(150)
		T2G	86.66 ± 145.06	60 (50)-(60)	162.64 ± 60.69	160 (120)-(200)	142.54 ± 57.37	140 (100)-(180)	131.07 ± 52.18	120 (100)-(150)	103.03 ± 44.83	100 (60)-(130)

^a^ = costs were calculated in Nepali rupees (NPR) [Exchange rate: 1 USD = NPR 73.38]

^b^ = CG = control group, T1G = test 1 group, T2G = test 2 group

^c^ = ADD = antidiabetic drug, AntiHTN = antihypertensive, others = Lipid lowering agents, Anti anginal drugs, Vitamin B complex, Antibiotic, Proton pump inhibitors, Antihistaminic, Antiplatelet agent

### Direct healthcare costs (direct medical + non-medical costs) of CG, T1G and T2G patients at baseline and follow-ups

The median direct medical costs, the median direct non-medical costs and the total median direct healthcare costs of CG, T1G and T2G patients at the baseline and follow-ups are mentioned in Table 2. The reduction in cost variables attributed to increased cost of patients could be achieved by successive counselling and diabetes education related to diabetes care from the pharmacist, which ultimately affected the direct medical and non-medical costs of patients resulting in a substantial reduction in total direct healthcare cost of patients in both test groups compared to control group in their follow-ups (Table 2).

**Table 2:** Total direct healthcare costs (direct medical and non-medical costs) of CG, T1G and T2G patients at the baseline and follow-ups

Follow-ups	Groups^b^	Direct medical costs^a^		Direct non-medical costs^a^		Total direct healthcare costs (Direct medical + non-medical costs)^a^	
		Mean cost ± sd	Median cost (IQR)	Mean cost ± sd	Median cost (IQR)	Mean cost ± sd	Median cost (IQR)
Baseline	CG	881.01 ± 1024.31	657 (445)-(879)	305.75 ± 557.60	129 (90)-(232)	1186.77 ± 1444.30	861 (583.75)-(1104.25)
	T1G	803.50 ± 455.06	737 (468)-(944.50)	225.22 ± 379.79	92.50 (80)-(200)	1028.72 ± 667.85	914 (615)-(1144)
	T2G	705.81 ± 450.93	567.50 (429)-(838)	188.98 ± 310.18	99 (80)-(150)	894.79 ± 660.23	712 (525.75)-(986.25)
3-months	CG	1926.62 ± 1226.31	1714.50 (1234.75)-(2176.25)	392.06 ± 211.27	320 (243.75)-(492.50)	2318.68 ± 1247.24	2057.50 (1645)-(2527.75)
	T1G	1990.96 ± 1180.94	1769 (1100)-(2452)	259.35 ± 80.69	240 (200)-(300)	2250.31 ± 1179.84	2071 (1350)-(2802)
	T2G	1911.37 ± 1003.66	1733 (1146)-(2358)	286.52 ± 111.37	300 (198)-(360)	2197.90 ± 1043.56	2062 (1384)-(2673)
6-months	CG	1875.56 ± 918.03	1712.50 (1235.50)-(2459)	301.78 ± 144.03	305 (218)-(360)	2177.34 ± 998.34	2080 (1467.25)-(2761.25)
	T1G	1621.58 ± 1220.84	1037 (738)-(2142)	247.13 ± 129.06	200 (150)-(310)	1868.72 ± 1217.24	1410 (925)-(2327)
	T2G	1512.45 ± 981.02	1309 (690)-(2020)	263.82 ± 119.56	255 (178)-(330)	1776.27 ± 1029.89	1543 (913)-(2263)
9-months	CG	1694.84 ± 730.40	1609.50 (1192.50)-(2098.25)	267.70 ± 90.34	275 (200)-(320)	1962.54 ± 767.27	1894.50 (1376.25)-(2378.50)
	T1G	1377.11 ± 723.19	1219 (753)-(1739)	226.74 ± 160.15	160 (128)-(320)	1603.86 ± 758.96	1374 (1104)-(1888)
	T2G	1276.74 ± 935.41	947 (631)-(1586)	251.17 ± 169.73	200 (148)-(300)	1527.92 ± 951.75	1260 (925)-(2144)
12-months	CG	1568.86 ± 565.84	1548 (1122.75)-(1866.75)	304.42 ± 92.58	307.50 (225)-(362.50)	1873.28 ± 587.99	1851.50 (1437.25)-(2272.25)
	T1G	1076.62 ± 726.61	768 (500)-(1488)	226.19 ± 126.02	184 (140)-(252)	1302.82 ± 771.70	1020 (660)-(1833)
	T2G	910.03 ± 650.91	620 (460)-(1116)	228.27 ± 193.20	180 (100)-(240)	1138.31 ± 682.27	900 (597)-(1455)

^a^ = costs were calculated in NPR [Exchange rate: 1 USD = NPR 73.38]

^b^ = CG = control group, T1G = test 1 group, T2G = test 2 group

### Direct healthcare costs comparison of patients at the baseline and follow-ups within test groups (T1G and T2G)

Friedman test identified the significant differences in DHCs of test 1 group (*p* < 0.001) and test 2 group (*p* < 0.001) patients due to pharmaceutical care intervention (Table 3).

**Table 3:** Direct healthcare costs comparison of patients at the baseline and follow-ups within test groups (T1G and T2G)

Groups	T1G (PC group)	T2G (PC + Diabetic kit group)
Follow-ups	Median cost (IQR)	Median cost (IQR)
Baseline	914 (615)-(1144)	712 (525.75)-(986.25)
3-months (1^st^ FU)	2071 (1350)-(2802)	2062 (1384)-(2673)
6-months (2^nd^ FU)	1410 (925)-(2327)	1543 (913)-(2263)
9-months (3^rd^ FU)	1374 (1104)-(1888)	1260 (925)-(2144)
12-months (4^th^ FU)	1020 (660)-(1833)	900 (597)-(1455)
***p*** -value*	<0.001**	<0.001**

*Friedman test applied

**Difference was significant at *p* ≤ 0.05 level (2-tailed)

However, it was difficult to explore from Friedman test where the actual significant differences occurred in each group on different occasions, which was resolved by using post-hoc analysis with the Wilcoxon signed rank test after Bonferroni adjustment applied (Table 4).

**Table 4:** Differences in direct healthcare costs of patients in both test groups (T1G and T2G) over time

Follow-ups	T1G (PC group)		T2G (PC + Diabetic kit group)	
	Z- value	*p*-value*	Z-value	*p*-value*
Baseline + 3-months	-5.352	<0.001**	-5.676	<0.001**
Baseline + 6-months	-4.396	<0.001**	-5.015	<0.001**
Baseline + 9-months	-4.134	<0.001**	-4.898	<0.001**
Baseline + 12-months	-2.132	0.033	-2.531	0.010
3-months + 6-months	-1.978	0.048	-2.235	0.025
3-months + 9-months	-4.134	<0.001**	-3.674	<0.001**
3-months + 12-months	-4.387	<0.001**	-5.408	<0.001**
6-months + 9-months	-1.322	0.186	-1.429	0.153
6-months + 12-months	-2.690	0.005**	-3.890	<0.001**
9-months + 12-months	-2.090	0.037	-4.218	<0.001**

* Wilcoxon signed rank test for paired data

**Difference was significant at *p* ≤ 0.005 level (2-tailed) after Bonferroni adjustment applied

### Comparison of direct healthcare costs between test groups (T1G and T2G), and CG and test groups’ patients

Although there were differences in median direct healthcare costs between the test groups (T1G and T2G) over time but differences were not statistically significant at Mann–Whitney *U*-test. Moreover, the significant differences in direct healthcare cost between CG and T1G, and T2G were noted at 6-months (*p* = 0.009, *p* = 0.010 respectively), 9-months (*p* = 0.005, *p* = 0.001 respectively) and 12-months (*p* < 0.001, *p* < 0.001 respectively) (Table 5).

**Table 5:** Comparison of direct healthcare costs between test groups (T1G and T2G) and, CG and test groups patients

Follow-ups	Baseline	3-months (1^st^ FU)	6-months (2^nd^ FU)	9-months (3^rd^ FU)	12-months (4^th^ FU)
Groups	Median cost (IQR)	Median cost (IQR)	Median cost (IQR)	Median cost (IQR)	Median cost (IQR)
T1G	914 (615)-(1144)	2071 (1350)-(2802)	1410 (925)-(2327)	1374 (1104)-(1888)	1020 (660)-(1833)
T2G	712 (525.75)-(986.25)	2062 (1384)-(2673)	1543 (913)-(2263)	1260 (925)-(2144)	900 (597)-(1455)
***p*** -value*	0.057	0.917	0.870	0.254	0.174
CG	861 (583.75)-(1104.25)	2057.50 (1645)-(2527.75)	2080 (1467.25)-(2761.25)	1894.50 (1376.25)-(2378.50)	1851.50 (1437.25)-(2272.25)
T1G	914 (615)-(1144)	2071 (1350)-(2802)	1410 (925)-(2327)	1374 (1104)-(1888)	1020 (660)-(1833)
***p*** -value*	0.463	0.642	0.009**	0.005**	<0.001**
CG	861 (583.75)-(1104.25)	2057.50 (1645)-(2527.75)	2080 (1467.25)-(2761.25)	1894.50 (1376.25)-(2378.50)	1851.50 (1437.25)-(2272.25)
T2G	712 (525.75)-(986.25)	2062 (1384)-(2673)	1543 (913)-(2263)	1260 (925)-(2144)	900 (597)-(1455)
***p*** -value*	0.260	0.405	0.010**	0.001**	<0.001**

* Mann–Whitney *U* test

**Difference was significant at *p* ≤ 0.05 level (2-tailed)

## Discussion

Diabetes is a very costly illness that creates a major impact on patient's direct healthcare costs (out-of-pocket expenses) [19]. The trend of increasing burden on patients’ out-of-pocket expenses is due to lack of patients’ focus on disease management in absence of their disease awareness and self-care practices.

The major contribution in total median direct healthcare costs of patients was attributed to median direct medical costs. The direct medical costs of patients in present study were greatly occupied with investigation costs followed by drug costs at baseline [5]. It was estimated that the total median direct healthcare costs of individual group at baseline was occupied by slightly more than half of median direct medical costs, which was further occupied by nearly one third of total median medication cost in each group. This is higher than the observation made from a study conducted in Italy [4]. Similarly, the dietary management costs and transportation costs were the main contributing components of direct non-medical costs at baseline in the present study.

Patients must be alert and proactive in managing their diabetes to avoid the co-morbidities that may cause significant out-of-pocket expenses of patients due to increased medical and medication costs related to diabetes and co-morbid conditions [19]. The out-of-pocket expenses of patients can be minimised by improving their health related outcomes. Pharmacist being an important member of healthcare team can provide a good support to patient in improving their health related outcomes and hence minimizing their out-of-pocket expenses. It is already evident from Asheville project in which authors demonstrated the impact of pharmaceutical care on economic outcomes in diabetes management [20]. There was significant increase in out-of-pocket expenses of patients in three groups at 3 months due to increase direct medical costs. The sudden increase in patients’ out-of-pocket expenses was due to the high degree of glycaemia that required frequent patient visits to the hospital during the first 3 months of the initiation phase of treatment to get the physician's consultation. This subsequently increased the costs of other components of direct medical and non-medical domains with major impact on drug costs, investigation cost, registration cost, transportation cost and dietary management cost. Together, this amplified the median direct healthcare costs of patients in the three groups at 3 months. A rise in out-of-pocket expenses may decrease or prevent the health-seeking behavior of the patients and prevent them from medication procurement [21].

Pharmacist led intervention reduced direct healthcare costs burden of patients significantly in test groups at 6, 9 and 12 months’ follow-ups compared to control group when tested by Mann–Whitney *U*-test. Similarly, few studies from USA also described a reduction in total direct medical costs and per patient direct healthcare cost due to pharmacist led intervention through pharmaceutical care program [20, 22, 23]. The major reduction was calculated in direct medical cost components such as investigation cost and prescription cost of patients [24]. This reduction could be due to improvement in glycaemic symptoms of patients that reduces their drug prescription and frequency of investigations. Moreover, a Colombian study also highlighted the reduction in medical cost of those patients who were under the care of pharmacist compared to control group patients [25].

The median DHCs of patients in test groups decreased significantly compared to control group due to reduction in their median direct medical costs at 12 month follow-up. Although, there were differences in median DHCs (in Nepali rupees) of patients in both test groups throughout the study period but it was not significant at any of the follow-ups. The insignificant decrease in median direct healthcare costs of patients in control group compared to test groups could be due to the doctor∕nurse provided care. The additional out-of-pocket expenses on patients in the absence of government and private health-insurance coverage^1^demands an urgent need of various healthcare schemes(e.g. health-insurance coverage) for the patients in developing countries like Nepal in order to protect their domestic budget, that may lead to improvement in their medication adherence and decrease the risk of chronic complications.

### Limitations of the study

Diabetes patients were selected from only one hospital of the Kaski district in western Nepal and hence the study findings may not be able to generalize to the entire diabetic population of the country. The study estimated the reduction in treatment costs but fail to analyse the decrease in number of drug (s) per prescription of patient after the intervention. Similarly, the decrease in number of patient visits to the hospital was also not accounted. Furthermore, cost of pharmacist services was not taken into account in present study as there was no such cost taken by the hospital from the patient where study was conducted.

## Conclusion

Direct healthcare costs of patients were mainly attributed to direct medical costs but contribution of transportation cost and dietary management cost cannot be ignored in direct non-medical cost. Pharmacist-provided intervention significantly decreased the direct healthcare costs of patients in test groups during their follow-ups with a greater reduction in drug costs and investigation costs. However, reduction in direct healthcare costs among control group patients was insignificant. The reduction in the direct healthcare costs of patients indicates the benefits of pharmacist-provided counselling and consultation through pharmaceutical care program in diabetes patient care and hence indicates pharmacist role and contribution in healthcare system.
